# Value of NSE and S100 Protein of Kawasaki Disease with Aseptic Meningitis in Infant

**DOI:** 10.1515/biol-2019-0040

**Published:** 2019-07-22

**Authors:** Jiangtao Wang, Shouhang Chen, Xiaoling Wang, Huiru Gu, Junli Liu, Xiaohong Wang, Liang Liu

**Affiliations:** 1 Department of Infant Ward, Children's Hospital Affiliated to Zhengzhou University, Henan Children’s Hospital, Zhengzhou Children’s Hospital, NO.33 Longhu East Road, Zhengzhou East District, Zhengzhou 450000, P.R. China

**Keywords:** Kawasaki disease, Aseptic meningitis, S100 protein, Neuron-specific enolase, Cerebrospinal fluid

## Abstract

The cerebrospinal fluid content was examined for concentrations of S100 protein and neuron-specific enolase (NSE) in two diseases, Kawasaki disease (KD) with aseptic meningitis (1-3 months) and purulent meningitis (PM), to determine whether or not these measuremets could be used in early diagnosis. The content of cerebrospinal fluid S100 protein of KD with aseptic meningitis and PM were significantly higher than those in the control group. There was also a difference between KD and purulent meningitis (PM). The concentration of NSE was highest in the encephalitis group, which was statistically different from control group. However, there was no difference between the KD and control groups. The levels of S100 protein and NSE of KD with aseptic meningitis were lower than those in PM, indicating that the extent of neuronal damage is significantly lower than of the enchephalitis group. The area under the curve (AUCs) of the receiver operating characteristic (ROC) curve for S100 and NSE were both 0.972. The S100 threshold was 0.4315, the sensitivity was 92.1%, and the specificity was 100%, while the NSE threshold was 9.325, sensitivity 92.1%, and specificity 90%. The combined detection of NSE and S100 levels in the cerebrospinal fluid can be used for the differential diagnosis of KD with aseptic meningitis and purulent meningitis.

## 1 Introduction

Kawasaki disease (KD), also known as mucocutaneous lymph node syndrome（MCLS）, is a systemic vasculitis characterized by the activation of the immune system and extensive damage to the endothelial cells, which can cause multiple organ damage and metabolic disorder [1,2]. Besides the heart, it can also damage the nervous system, showing as aseptic meningitis [3]. KD with neurologic complications is uncommon. Aseptic meningitis often occurs in the acute stage, and manifested mainly as increased intracranial pressure, resulting in symptoms such as headache, vomiting, anterior fontanelle bulge, and meningeal irritation, and seizures can occur in some cases. On routine inspection of CSF, increased white blood cells, particularly lymphocytes are observed. Sugar and chloride are usually normal, and protein can be slightly increased and may be accompanied by electroencephalogram abnormalities. If the above clinical manifestations are present, the diagnosis of viral meningitis can be established [4]. KD combined with aseptic meningitis results in a diversity of the clinical manifestations, especially during the acute phase. Infancy is also a high onset age for purulent meningitis. KD can also be characterized by the nervous system damage and aseptic meningitis in the early stages of the disease, without obvious lesions of the skin and mucous membrane, so it is easily misdiagnosed as an atypical purulent meningitis [5]. Early diagnosis and management of Kawasaki disease are important to ensure a good outcome and a high index of suspicion in febrile children is required irrespective of the clinical presentation[6]. In order to improve the understanding of the disease and reduce misdiagnosis, a retrospective analysis of the cerebrospinal fluid S100 protein and NSE was processed, based on the diagnostic result of the children who had either Kawasaki disease with aseptic meningitis or the purulent meningitis, between January 2014 and December 2015 in our hospital. The purpose was to provide the basis for early differential diagnosis.

## 2 Subjects and methods

### 2.1 Patients

The clinical data from 21 children with Kawasaki disease combined with aseptic meningitis and 38 cases of purulent meningitis at the Zhengzhou children’s Hospital from January 2014 to December 2015 were collected. The control group consisted of 20 children with a fever for more than 3 days, suspected infection of the central nervous system, and lumbar puncture was negative. The lumbar puncture operation should be performed 7 days before the course of the disease, before injection of gamma globulin, and cerebrospinal fluid is clear (CSF red cells < 1000 /mm^3^). Among the 21 children with KD combined with sterile meningitis, 13 were male and 8 were female, with an average age of 74.381±24.117d. Of the 38 children with PM, 23 were male and 15 were female, with an average age of 88.447±30.041d; among the 20 fever control groups, 11 were male and 9 were female, with an average age of 83.468±27.687d; There was no significant difference in age and gender distribution between the three groups (P = 0.05) (Table 1 and 2).

**Table 1:** The result of gender of three groups

Group	male	female
KD Group	13	8
PM Group	23	15
Control Group	11	9
c2	0.235*	

Note：*P=0.88＞0.05

**Table 2:** The result of age in days of three groups

Group	Age in days	P
KD Group	74.381±24.117	0.004#
PM Group	88.447±30.041	
Control Group	100.45±29.337	

Note：#KD Group compares with Control Group： P=0.004<0.05

### 2.2 Clinical features

The clinical manifestations of KD combined with sterile meningitis were in the acute phase of disease (≤10d) (Table 3).

**Table 3:** The clinical manifestations of KD with sterile meningitis

case	clinical manifestations	coronary expansion
**1**	Fever, erythema,bilateral conjunctival hyperemia,	yes
**2**	Fever, lip chapped, bilateral conjunctival hyperemia, erythema, lymphadenopathy	yes
**3**	Fever, lymphadenopathy, bilateral conjunctival hyperemia, erythema, lip redness and chapped	no
**4**	Fever, lymphadenopathy, bilateral conjunctival hyperemia, erythema, lip redness and chapped	no
**5**	Fever, erythema, bilateral conjunctival hyperemia, lip redness and chapped	yes
**6**	Fever, lymphadenopathy, lip redness and chapped	yes
**7**	Fever, lip redness, lymphadenopathy, bilateral conjunctival hyperemia,	yes
**8**	Fever, hard edema of the hands and feet; bayberry tongue, erythema, bilateral conjunctival hyperemia	no
**9**	Fever, oral and pharyngeal mucosa diffuse hyperemia, lip chapped, erythema bayberry tongue lymphadenopathy	no
**10**	Fever, hard edema of the hands and feet, bayberry tongue	yes
**11**	Fever, lymphadenopathy, bilateral conjunctival hyperemia, erythema, hard edema of the hands and feet	no
**12**	Fever, bilateral conjunctival hyperemia，erythema, hard edema of the hands and feet, membranous peeling of the	no
	finger tip oral and pharyngeal mucosa diffuse hyperemia, lip chapped,	
**13**	Fever, hard edema of the hands and feet, membranous peeling of the finger tip oral and pharyngeal mucosa diffuse hyperemia	yes
**14**	Fever, lip chapped, bayberry tongue， lymphadenopathy， bilateral conjunctival hyperemia， erythema	no
**15**	Feverv, bayberry tongue, lip chapped, lymphadenopathy， bilateral conjunctival hyperemia， erythema	no
**16**	Fever, lip chapped, bayberry tongue, lymphadenopathy, bilateral conjunctival hyperemia, erythema	no
**17**	Fever, hard edema of the hands and feet, bayberry tongue, Lymphadenopathy, bilateral conjunctival hyperemia	no
**18**	Fever, lip chapped, lymphadenopathy bilateral conjunctival hyperemia erythema	no
**19**	Fever, hard edema of the hands and feet, bilateral conjunctival hyperemia, lip chapped, erythema	yes
**20**	Fever, bilateral conjunctival hyperemia, lip chapped, erythema	yes
**21**	Fever, oral and pharyngeal mucosa diffuse hyperemia, lip chapped, bayberry tongue, lymphadenopathy, bilateral conjunctival hyperemia, erythema	no

### 2.3 Diagnostic Criteria

The main diagnostic criteria for Kawasaki disease were based on the diagnostic criteria established by the Kawasaki Disease Research Commission of Japan in 1984: (1) fever time lasts for more than 5 days; (2) bilateral conjunctival hyperemia; (3) lip congestion and cleft palate, diffuse congestion of oral mucosa, tongue nipple, congestion of strawberry tongue; (4) polymorphous erythema; (5) hard edema of the hands and feet, membranous peeling of the toe end during recovery; (6) acute non-suppurative cervical lymphadenopathy. Kawasaki disease can be diagnosed if 5 of these 6 diagnostic criteria are met, or, if there are less than 5, the additional presence of echocardiographic coronary artery lesions.

The diagnosis of coronary artery dilatation is based on the diagnostic criteria of Tsai. [7]. (1) Coronary artery abnormalities: Coronary artery diameter ≥3 mm in patients <5 years old, coronary artery diameter ≥4 mm in patients ≥5 years old. The ratio of coronary artery to aortic root inner diameter (CA/AO) <0.3; (2) coronary aneurysm: coronary artery diameter ≥ 8 mm, CA / AO = 0.3.

Diagnostic criteria for aseptic meningitis [8]: White blood cell counts in CSF = 15/mm^3^ for children aged 4-8 weeks; white blood cell counts in CSF = 7/mm^3^ for children age = 8 weeks.

Purulent meningitis was diagnosed according to diagnostic criteria of “Zhu Futang Practical Pediatrics eighth edition” [9]. Clinical fever, headache, vomiting, mental changes and meningeal irritation and CSF changes; CSF and/or blood bacterial culture positive, or CSF smears detect pathogens; for pathogenic detection negative cases, a peripheral blood white blood cell count =10×109/L, mainly neutrophils, was required. Additionally, the cerebrospinal fluid white blood cell count was =500×106/L, neutrophil dominant, and CSF protein content was increased by ≥100 mg/dl.

### 2.4 Method

The contents of S100 protein and NSE in cerebrospinal fluid were determined by enzyme-linked immunosorbent assay (ELISA) using a kit provided by Shanghai West Tang biological Co., Ltd. The operation steps are strictly carried out in accordance with the instructions. Cerebrospinal fluid white blood cell count, protein, sugar, and chloride determination were provided by the laboratory department of our hospital, according to the operation standard.

### 2.5 Statistical Analysis

The data were analyzed using SPSS19.0 statistical software. The data were expressed by `x± s, and the single factor analysis of variance was carried out. The data between each of the two groups were compared with the LSD method, and the difference of P< 0.05 was statistically significant. The receiver operating characteristic( ROC) was illustrated and the area under the curve (AUC) calculated.

## 3 Results

The diagnostic results for the three groups concerning concentrations of S100 protein and NSE in the cerebrospinal fluid were as follows: the content of cerebrospinal fluid S100 protein of KD with aseptic meningitis and PM were significantly higher than those in the control group (P<0.05). There was also a difference between KD and PM (P<0.05). The content of NSE was highest for the PM group and was statistically different from the control group (P<0.05). There was no difference between the KD and the control groups (P=0.05)（Table 4）.

**Table 4:** The diagnostic results of three groups of S100 protein and NSE in cerebrospinal fluid (ng/ml)

Group(cases)	S100 protein	NSE
Control group(20)	0.043±0.025#	6.518±1.536
KD group(21)	0.288±0.107*	7.358±1.461
PM group(38)	0.569±0.117*#	12.1±1.881*#
F	191.777	92.356

Note: Compared with the control group, * P<0.05;compared with the KD group, # P < 0. 05

**Figure 1:**
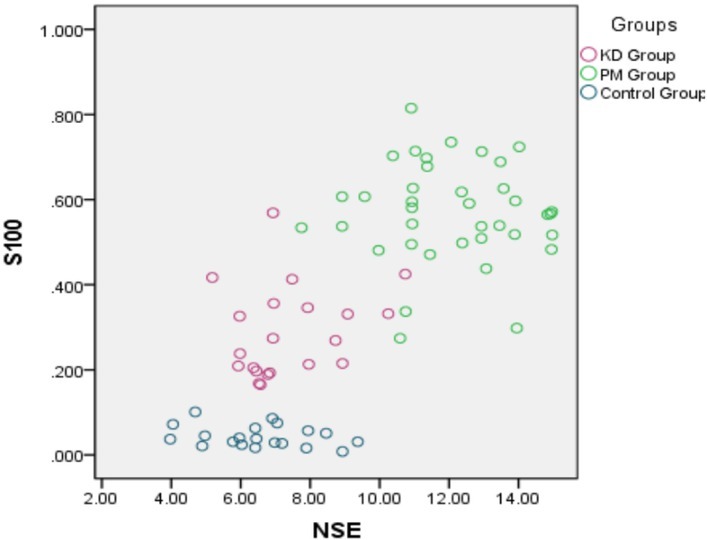
The scattered diagram of individual results

The results for each group are depicted in Figure 1.

**Figure 2.1 - 2.2:**
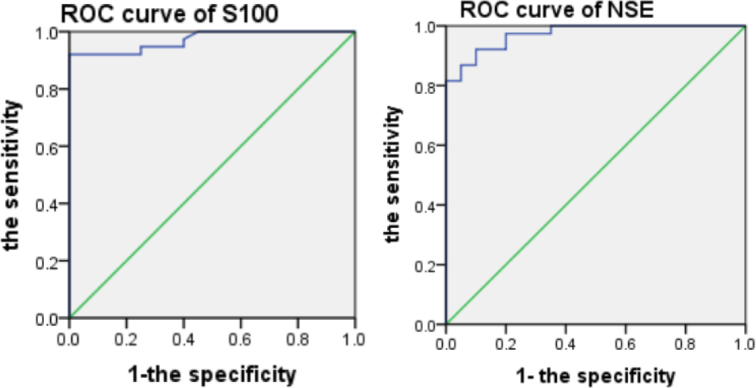
Note: AUCs of the ROC curve of S100 and NSE are both 0.972.

The ROC curve analysis for S100 and NSE in the cerebrospinal fluid for the three groups determined that AUCs of the ROC curve were both 0.972. The sensitivity and specificity of the diagnosis was claucalted using the critical values of the Yoden Index. The results showed that the S100 threshold was 0.4315, the sensitivity was 92.1%, and the specificity was 100%, while the NSE threshold wass 9.325, sensitivity 92.1%, specificity 90% (Table 5, Figure 2.1-2.2).

**Table 5:** The results of ROC curves of S100 and NSE in CSF for the three groups

Indicators	AUC	Sensitivity	Specificity	Standard error
S100	0.972	92.1%	100%	0.018
NSE	0.972	92.1%	90%	0.017

## 4 Discussion

Cerebrospinal fluid S100 protein and NSE are considered as markers of glial cell and neuronal damage [10]. S100 protein is a type of calcium ion binding protein and is a growth factor of the axon. It is mainly distributed in the astrocytes and Schwann cells of the central nervous system (CNS) and the peripheral nervous system (PNS). It is a bridge of interaction between glia and neurons. S100 protein acts on the neuron and its surrounding environment and is specific to the nervous system. As a result of the integrity of brain cells and blood brain barrier, the level of S100 in the blood and cerebrospinal fluid is typically very low in healthy people, but due to some diseases,Traumatic brain injury (TBI) or some other causes (such as cerebrovascular hemorrhage, viral encephalitis, child concussion, neonatal hypoxia, brain tumor, neuroinflammation, neurodegenerative changes, etc.) the S100 protein can be released from cells andenter the cerebrospinal fluid or blood through the damaged blood brain barrier. Thereby, increasing the concentration of S100 in the blood and cerebrospinal fluid [11]. Brain injury increases significantly , and the amount of S100 protein correlates to the degree of brain injury. NSE mainly exists in the cytoplasm of central nervous system neurons and neuroendocrine cells. Under normal circumstances, it has a very low concentration in bodily fluids. In the case of brain injury, the nerve cells disintegrate and are destroyed, the enzyme enters the cerebrospinal fluid and blood, increasing its level which can reflect the extent of the CNS damage. It is a very sensitive and specific marker of neuronal damage [12, 13].

This study shows that the concentration of S100 protein in cerebrospinal fluid of children with purulent meningitis is significantly higher than that of children with aseptic meningitis or controls. The S100 protein of cerebrospinal fluid in the children with aseptic meningitis is also higher than that in the control group. This suggests that the neuroglial cells are damaged in the children with KD with aseptic meningitis, but the damage to the suppurative meninges is slight. So it is considered that KD with aseptic meningitis may be associated with cerebral microvasculitis and brain edema, leading to inadequacy of perfusion with local cerebral blood flow, thereby causing damage tothe Neuroglial cells. The levels of S100 protein and NSE in infant KD with aseptic meningitis were lower than those in the purulent meningitis group, indicating that the extent of neuronal damage was significantly lower than that of the encephalitis group.

A ROC curve is a combination of sensitivity and specificity for two indicators, to objectively evaluate their accuracy in diagnosis. The results of this study showed that the area under the ROC curve for NSE was 0.972. The area under the ROC curve of S100 was 0.972, suggesting that NSE and S100 combined detection of KD with aseptic meningitis has the high diagnostic value.

The early diagnosis and identification of Kawasaki Disease with aseptic meningitis and the correct treatment will help to reduce the risk of coronary artery dilatation or even coronary artery aneurysm.This is practical, as these diseases are often difficult to diagnosis during the acute stage.

The results of this study show that the determination of S100 protein and NSE content in the cerebrospinal fluid not only can identify damage of glial cells and neurons, but also provide objective clinical indicators for the identification of Kawasaki disease with aseptic meningitis and suppurative meningitis.
